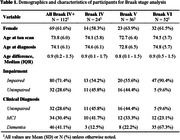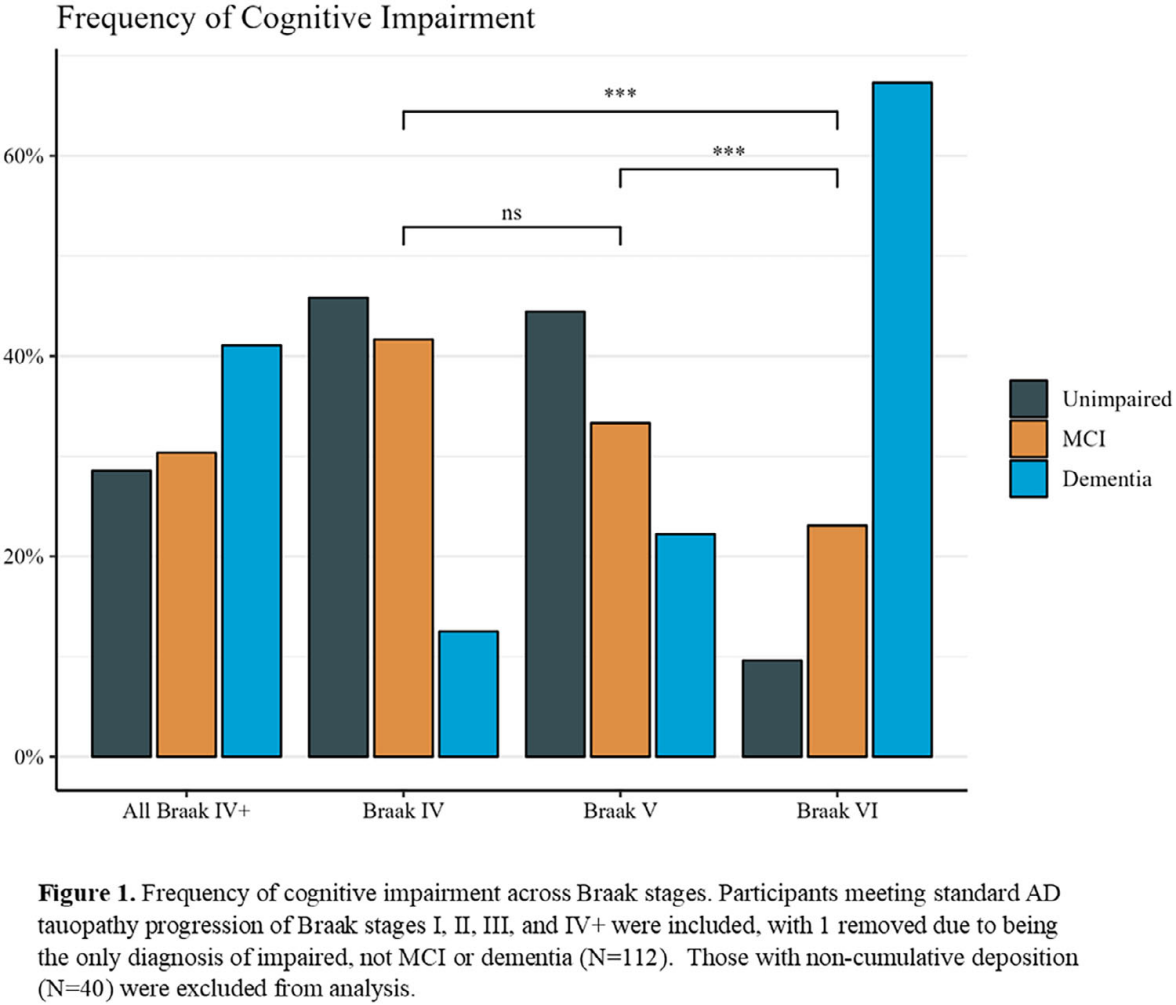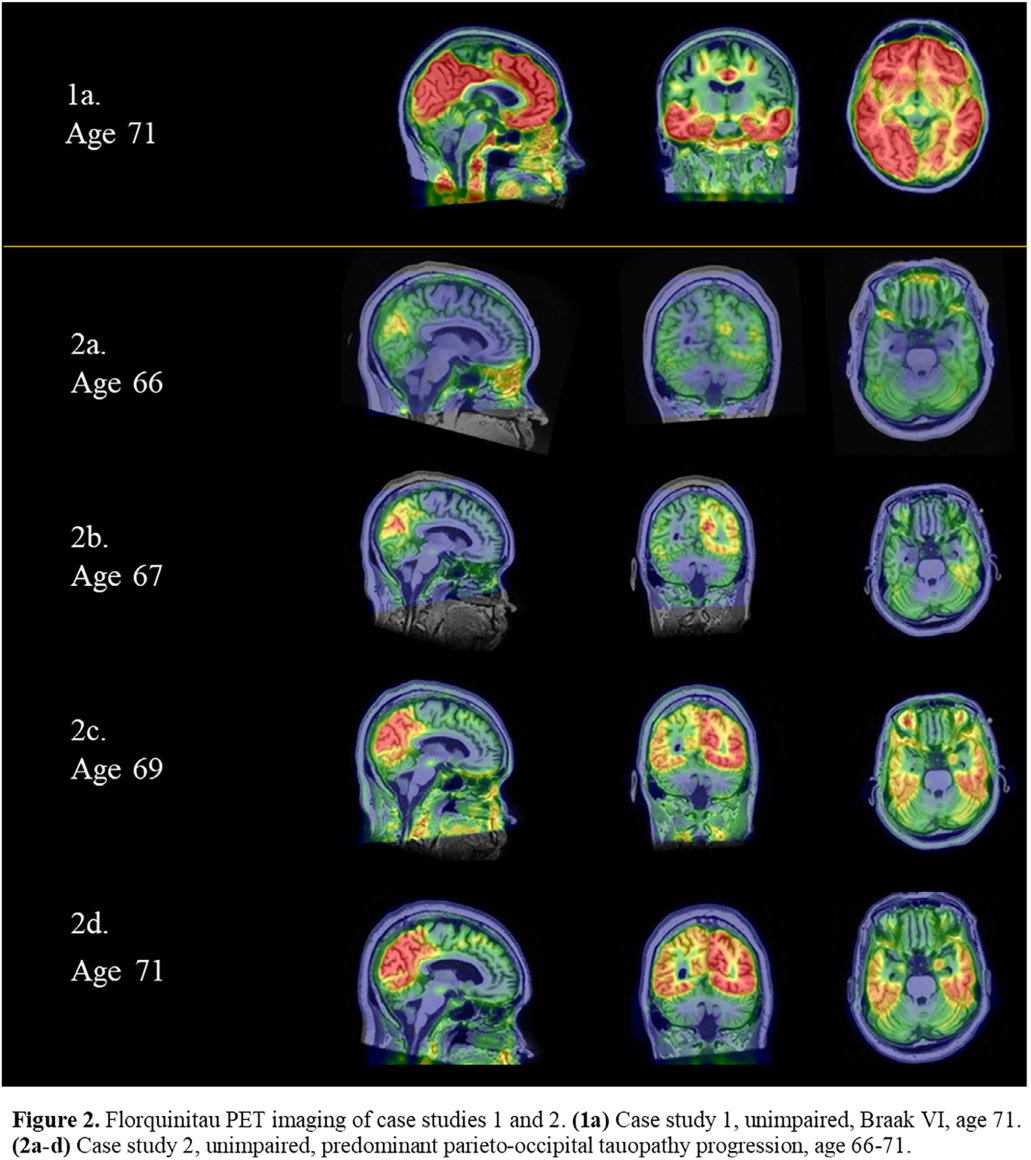# Cognitive outcomes with AD tauopathy characterized by florquinitau PET

**DOI:** 10.1002/alz.092841

**Published:** 2025-01-09

**Authors:** Melissa Bahr, Madilynn Wintlend, Olivia R Mandel, Rachel L Studer, Erin M. Jonaitis, Sara Frost Alberson, Emma Meyering, Brenda Ryther, Teresa Hellenbrand, Noelle Kaminski, Nathaniel A. Chin, Tobey J. Betthauser, Bradley T. Christian, Sterling C. Johnson

**Affiliations:** ^1^ University of Wisconsin School of Medicine and Public Health, Madison, WI USA; ^2^ University of Wisconsin‐Madison School of Medicine and Public Health, Madison, WI USA; ^3^ Department of Medicine, University of Wisconsin‐Madison School of Medicine and Public Health, Madison, WI USA

## Abstract

**Background:**

PET imaging studies examining amyloid‐β plaques and tau neurofibrillary tangles, concurrent with cognitive testing, give insight into in vivo Alzheimer’s Disease (AD) pathology burden and corresponding clinical outcomes. Braak stages IV‐VI indicate advanced tau aggregation in the association and primary cortices associated with AD neuropathological changes which are often accompanied by cognitive decline or impairment. Not all individuals with AD pathological changes present with the expected rate of cognitive decline or standard Braak staging progression, such as in cases of resilience or posterior cortical atrophy (PCA), a variant of AD identified by parieto‐occipital tau aggregation.

**Methods:**

Tau PET data were obtained from two Wisconsin Alzheimer’s disease cohorts (N = 830). Participants were assigned to a Braak stage if they were positive for tau in that region and all prior regions (cumulative deposition), and those in Braak stages IV‐VI (N = 112, Table 1) were included. Chi‐square tests were completed to examine the relationship between Braak staging and concurrent research diagnoses. Participants with non‐cumulative deposition were excluded (N=40); two with non‐AD‐like patterns are described.

**Results:**

Of the research participants with a tau PET scan rated Braak IV or higher, 71.4% were impaired (Figure 1). Diagnoses differed significantly across Braak Stages, χ^2^ (4, N = 112) = 30.8, p = .00050. Specifically, Stage VI differed from IV (p < .001) and V (p < .001).

We present 2 individual case studies of participants who deviate from this pattern (Figure 2). Case study 1 presented without cognitive impairment, despite tau PET scans rated as Braak VI. Case study 2 presented without cognitive impairment and predominant parieto‐occipital tau burden suggestive of PCA. The tau depositions in the presumed PCA case initially localized to the medial occipital region and spread in reverse hierarchical order of tauopathy in all regions over 5 years.

**Conclusion:**

This analysis of tau PET data supports a relationship between the visual read estimate of tau burden and cognitive change. Continued research on the heterogeneity of disease progression, such as presented in these case studies, is important to further our understanding of disease development, resilience factors, and clinically relevant outcomes.